# The Effectiveness of Sacubitril/Valsartan in Systemic Sclerosis Patients with Heart Failure: A Retrospective Analysis

**DOI:** 10.3390/jcm14124054

**Published:** 2025-06-08

**Authors:** Nouran Eshak, Mahmoud Abdelnabi, Jaxon Quillen, Micheal Pham, Joseph Hentz, Vivek Nagaraja

**Affiliations:** 1 Division of Rheumatology, Department of Internal Medicine, Mayo Clinic, Phoenix, AZ 85054, USA; eshak.nouran@mayo.edu (N.E.); pham.micheal@mayo.edu (M.P.); 2 Department of Cardiovascular Medicine, Mayo Clinic, Phoenix, AZ 85054, USA; abdelnabi.mahmoud@mayo.edu; 3 Department of Biostatistics, Mayo Clinic, Scottsdale, AZ 85259, USA; quillen.jaxon@mayo.edu (J.Q.); hentz.joseph@mayo.edu (J.H.)

**Keywords:** sacubitril/valsartan, systemic sclerosis, heart failure with reduced ejection fraction, cardiovascular outcomes

## Abstract

**Introduction:** Cardiac involvement in patients with systemic sclerosis (SSc) can present variably from being asymptomatic to manifesting with heart failure, conduction abnormalities, pulmonary hypertension, and pericardial effusion. Symptomatic cardiac involvement portends a poor prognosis and worse overall survival. Sacubitril/valsartan (SV), an angiotensin receptor neprilysin inhibitor, has been shown to significantly reduce hospitalization rates and morbidity in patients with heart failure with reduced ejection fraction (HFrEF). This study aimed to investigate the effects of SV treatment in patients with SSc and heart failure. **Methods:** A retrospective analysis of patients with SSc was conducted using an electronic data capture tool. Patients with SSc treated with SV between January 2015 and August 2023 were identified. Comprehensive clinical phenotyping and longitudinal data analysis were performed to characterize the sub-type of patients and evaluate clinical outcomes, including hospitalizations and mortality, laboratory markers, and echocardiographic findings. **Results:** Twenty-four patients with SSc were treated with SV for a mean duration of 20.6 months. HFrEF was the primary indication for SV use in 91% of patients, primarily due to non-ischemic cardiomyopathy (87.5%). There was a significant reduction in systolic blood pressure from 128 mmHg to 114 mmHg (*p* < 0.001) and NT-proBNP levels from 15,130 pg/mL to 5082 pg/mL (*p* = 0.046). In the 19 patients with baseline and follow-up echocardiograms, there was a significant improvement in LVEF from 40.3% to 47.7% (*p* = 0.014). Hypotension was a common side effect leading to discontinuation of SV (n = 4, 16.7%). Serum creatinine had trends of improvement (1.9 mg/dL to 1.3 mg/d), though it did not reach statistical significance (*p* = 0.057). **Conclusions:** This study showed that SV effectively improved cardiac symptoms and function in patients with SSc presenting with HFrEF. Further prospective studies are needed to confirm these findings and explore the role of SV in the treatment of other manifestations of SSc.

## 1. Introduction

Systemic sclerosis (SSc) is a complex systemic autoimmune disease with an overlapping and contiguous pathophysiologic process characterized by vasculopathy, immune dysregulation, and progressive fibrosis [1]. Commonly involved systems include the skin, gastrointestinal tract, lungs, kidneys, and heart. Cardiac involvement in SSc is characterized by a spectrum of primary and secondary cardiac manifestations, most notably myocardial fibrosis, diastolic and systolic dysfunction, arrhythmias, conduction disturbances, pericardial disease, and, less commonly, valvular abnormalities and coronary microvascular disease [2,3]. Cardiac involvement in SSc is usually associated with significant morbidity and mortality [3,4]. The prevalence of clinically evident cardiac involvement in SSc is approximately 10–15% of SSc patients at baseline in large cohort studies, with a 5-year cumulative incidence of about 11% for new cases. The American College of Rheumatology and the European Alliance of Associations for Rheumatology (ACR/EULAR) note that the incidence of specific cardiac complications ranges from 10 to 32% in diffuse cutaneous SSc (dcSSc) and 12 to 23% in limited cutaneous SSc (lcSSc), but subclinical involvement is much higher; nearly all patients may have some degree of myocardial fibrosis detectable by advanced imaging, even in the absence of symptoms [5,6]. Myocardial fibrosis, driven by microvascular damage and persistent inflammation, is a hallmark of cardiac SSc and underpins many of its clinical manifestations. Advances in cardiac imaging modalities, including strain echocardiography and cardiac magnetic resonance (CMR), have further highlighted the presence of myocardial fibrosis. Studies utilizing CMR have demonstrated characteristic patterns of late gadolinium enhancement, often reflecting widespread subendocardial fibrosis in non-coronary artery territories, indicative of primary SSc-related cardiac involvement [7]. Cardiac involvement in SSc is associated with a poor prognosis, with myocardial fibrosis and diastolic dysfunction being key predictors of mortality [8,9]. Sacubitril/valsartan (SV), a first-in-class angiotensin receptor–neprilysin inhibitor (ARNI), has significantly reduced cardiovascular mortality and hospitalizations in patients with HFrEF [10]. Sacubitril inhibits neprilysin, an enzyme responsible for degrading natriuretic peptides, bradykinin, and other vasoactive substances, enhancing their effects such as vasodilation, natriuresis, and anti-remodeling. Valsartan, an angiotensin II receptor blocker, counteracts the renin–angiotensin–aldosterone system, reducing vasoconstriction, sodium retention, and adverse cardiac remodeling [10]. Previous data suggest that the antifibrotic actions of SV are mediated by direct effects on cardiac fibroblasts, the inhibition of profibrotic signaling, and favorable modulation of extracellular matrix homeostasis, which contribute to its clinical benefits in heart failure and hypertensive populations [11,12]. In extrapolating these mechanistic effects, SV could be a therapeutic option in SSc-related heart failure, potentially attenuating myocardial fibrosis and improving cardiac function by addressing the fibrotic and hemodynamic pathways [13].

This study aimed to investigate the effect of SV on a cohort of patients with SSc and heart failure.

## 2. Materials and Methods

### 2.1. Study Population

This retrospective cohort study was conducted using data extracted from the electronic health record (EHR) systems across all Mayo Clinic sites (Arizona, Florida, Minnesota, and associated health system sites). A systematic search was conducted using ICD-9 (710.1) and ICD-10 (M34.x) diagnostic codes to identify patients with systemic sclerosis (SSc), also known as scleroderma, who had been prescribed sacubitril/valsartan (SV) between January 2015 and August 2023. In addition to structured code-based searches, a natural language processing (NLP)-enabled text search algorithm was employed to capture unstructured documentation containing terms such as “scleroderma,” “systemic sclerosis,” “sacubitril/valsartan,” or “Entresto” within clinical notes and medication histories.

Patients were included if they met the following criteria: (1) age ≥18 years at the time of SV initiation; (2) a confirmed diagnosis of SSc based on the 2013 American College of Rheumatology/European Alliance of Associations for Rheumatology (ACR/EULAR) classification criteria; and (3) documented use of sacubitril/valsartan for a minimum duration of one month. Exclusion criteria included patients with incomplete records, uncertain SSc diagnosis, or those who discontinued SV within the first month due to adverse effects or intolerance.

### 2.2. Data Collection

Data collection was performed using REDCap (Research Electronic Data Capture), a secure, web-based software platform designed to support data capture for research studies. The dataset included both baseline and follow-up clinical variables. Demographic information included age, sex, and various comorbidities. Detailed clinical phenotyping of SSc, duration of disease prior to SV initiation, and overlap features with other connective tissue diseases were also included.

Cardiac involvement was defined based on the presence of heart failure symptoms, echocardiographic abnormalities, elevated natriuretic peptides, or cardiac MRI findings consistent with myocardial fibrosis or inflammation. Details related to sacubitril/valsartan treatment were recorded, including initiation date, dosing, titration schedule, concomitant heart failure therapies, and any documented adverse events.

Outcomes of interest included changes in New York Heart Association (NYHA) functional class, blood pressure, echocardiographic parameters (particularly left ventricular ejection fraction [LVEF]), and biomarkers such as N-terminal pro–B-type natriuretic peptide (NT-proBNP). Additional outcomes included hospitalizations for heart failure and overall survival. Data on renal function, electrolyte abnormalities, and follow-up time were also collected. Follow-up was censored at the date of last contact, death, or 31 August 2023.

### 2.3. Statistical Analysis

Descriptive statistics were used to summarize the baseline characteristics of the study cohort, with continuous variables described as means ± standard deviation. Paired *T*-tests were used to compare baseline and follow-up values for continuous variables, including changes in blood pressure, LVEF, and NT-proBNP levels. Independent *t*-tests were used to assess differences between SSc subtypes, and chi-square tests were used for categorical data. Kaplan–Meier survival analysis was performed to estimate time to hospitalization and overall survival rates.

## 3. Results

### 3.1. Cohort Selection and Characteristics

A total of 99 unique patients were initially identified from the comprehensive database search, which included all charts containing any of the following ICD codes for systemic sclerosis: M34.0, M34.1, M34.2, M34.8, M34.81, M34.82, M34.83, M34.89, M34.9, and L94.0, in combination with a medication order for SV. During the first round of manual chart review, 59 patients were excluded because no documented clinical diagnosis of SSc was found, despite the presence of an ICD code. An additional seven patients were excluded because SV therapy was administered for less than 4 weeks, making it unlikely that meaningful clinical outcomes could be attributed to treatment. Furthermore, nine patients were excluded from the cohort because, although they had an underlying autoimmune disease, they did not meet the 2013 ACR/EULAR classification criteria for SSc. This screening process resulted in a final study cohort of 24 patients with confirmed SSc and at least 4 weeks of SV treatment.

### 3.2. Demographics and Baseline Clinical Characteristics

The final cohort had a female predominance (75%), with a mean age of 53 years (SD ± 13) at the time of SSc diagnosis. The mean duration of disease prior to SV initiation was 6.8 years (SD ± 5.6). Clinical phenotyping revealed that limited cutaneous SSc was the most common subtype (n = 11, 45%), followed by diffuse cutaneous SSc (n = 9, 37%), with smaller proportions of overlap (n = 2, 8%) and sine scleroderma (n = 1, 4%).

The average duration of SV use was 20.6 months (SD ± 20), and the primary indication was heart failure with reduced ejection fraction (HFrEF) in 87.5% (n = 21), mostly in the context of non-ischemic cardiomyopathy. SV was prescribed at a dose of 24/26 mg in 70.8% of patients. Discontinuation occurred in six patients (25%), with hypotension-related symptoms (e.g., dizziness, lightheadedness) being the most common cause (16%) and mortality (8%).

### 3.3. Functional, Laboratory, and Hemodynamic Outcomes

**Figure 1:**
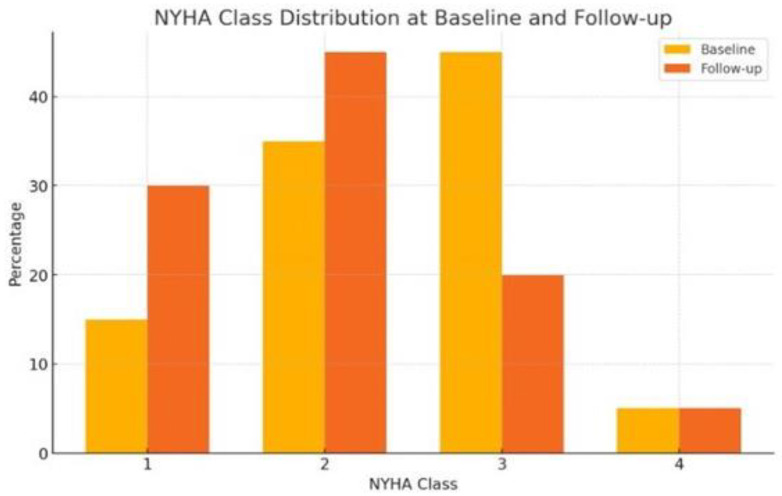
Comparison between NYHA class distribution at baseline and follow-up.

SV therapy was associated with significant improvements in several clinical outcomes. At baseline, the distribution of patients by NYHA functional class was as follows: Class I (15%), Class II (35%), Class III (45%), and Class IV (5%). After treatment with SV, there was a shift toward lower NYHA classes: Class I (30%), Class II (45%), Class III (20%), and Class IV (5%) (*p* = 0.04), indicating improved heart failure symptoms (Figure 1). Systolic blood pressure significantly decreased from a mean of 128 mmHg to 114 mmHg (Δ-14 mmHg; 95% CI: −20.1 to −8.4; *p* < 0.001). NT-ProBNP levels decreased substantially from 15,130.1 pg/mL to 5082.4 pg/mL (Δ-10,047.7 pg/mL; 95% CI: −19,864.1 to −231.3; *p* = 0.046). Creatinine levels decreased from 1.9 to 1.3 mg/dl, though this was not statistically significant (*p* = 0.057). Mean left ventricular ejection fraction (LVEF) improved from 40.3% to 47.7% (Δ7.4%; 95% CI: 1.7 to 13.2; *p* = 0.014) (Table 1 and Table 2). Right ventricular systolic pressure (RVSP) decreased modestly (Δ-3.9 mmHg), although this was not statistically significant (*p* = 0.202). Creatinine levels showed a downward trend from 1.9 mg/dL to 1.3 mg/dL, although this change was not statistically significant (*p* = 0.057).

### 3.4. Subgroup Comparison by SSc Phenotype

No statistically significant differences were observed in sex distribution, age at diagnosis, SV treatment duration, or discontinuation rates. Both groups demonstrated improvement in LVEF over time, with similar five-year survival rates (Table 3).

### 3.5. Survival Time and Hospitalization

**Figure 2:**
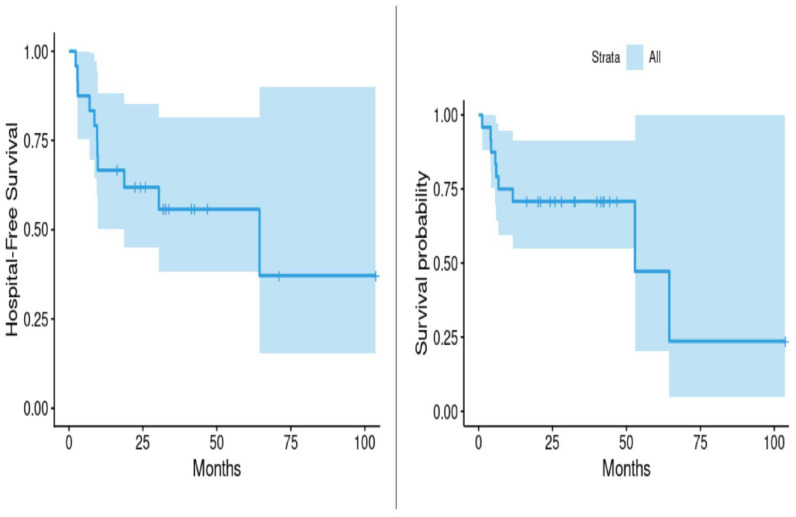
Hospitalization and survival following treatment with sacubitril/valsartan for scleroderma.

Using Kaplan–Meier estimates, the median survival time following initiation of SV therapy was 52.9 months, while the median time to first hospitalization due to any cause was 64.4 months. These data suggest a prolonged event-free period among SSc patients treated with SV, despite the chronicity and complexity of their disease (Table 2, Figure 2).

## 4. Discussion

This retrospective study demonstrated that SV was associated with significant improvements in heart failure symptoms and cardiac function among patients with SSc. Specifically, patients showed a reduction in NT-proBNP levels, improvement in LVEF, and a decrease in NYHA functional class. Furthermore, renal function remained stable throughout the study period, showing the potential safety of SV in SSc. Additionally, a modest decrease in RVSP was noted, indicating a potential improvement of RV systolic function; however, it did not reach statistical significance, likely due to sample size limitations or heterogeneity in pulmonary involvement among patients with SSc. These findings suggest that SV can be associated with clinical benefits in this high-risk population.

To date, the literature remains limited regarding the use of SV in SSc-specific heart failure, particularly in the context of myocardial fibrosis and mixed cardiac phenotypes (e.g., overlap of preserved and reduced ejection fraction). Most existing data are extrapolated from general heart failure populations [14]. In our study, the average 7% increase in LVEF and corresponding reduction in NT-proBNP are consistent with those observed in broader heart failure with reduced ejection fraction (HFrEF) cohorts. For instance, in the PROVE-HF study by Januzzi et al., patients receiving SV had an LVEF increase from 28.2% to 37.8% and a median NT-proBNP reduction from 816 pg/mL to 455 pg/mL over 12 months, highlighting the therapy’s favorable effects on cardiac remodeling [15].

SV’s therapeutic effect is mediated through dual inhibition: Sacubitril inhibits neprilysin, leading to enhanced levels of endogenous natriuretic peptides, while valsartan blocks the angiotensin II type 1 receptor. This dual action increases intracellular cyclic guanosine monophosphate (cGMP), activating protein kinase G, which in turn promotes vasodilation, natriuresis, the inhibition of maladaptive neurohormonal activation, and the attenuation of myocardial fibrosis, hypertrophy, and apoptosis [16]. These molecular changes support reverse remodeling and improve myocardial performance, which are particularly relevant in SSc, where cardiac involvement is often driven by diffuse fibrosis and microvascular dysfunction [16].

Furthermore, several clinical trials have shown that SV can cause reverse remodeling and improve outcomes in both ischemic and non-ischemic cardiomyopathy [17]. Since its FDA approval in 2015, SV has been incorporated into the guideline-directed medical therapy (GDMT) for treating patients with HFrEF by the American Heart Association.Its benefits extend beyond left ventricular systolic function: Recent studies have also demonstrated reductions in mean pulmonary arterial pressure (mPAP) in patients with heart failure with preserved ejection fraction (HFpEF) and pulmonary hypertension [16,18]. This observation can be particularly beneficial in SSc patients, who frequently have pulmonary vascular involvement, and may suggest broader therapeutic indications for SV in this population.

Additionally, previous studies have highlighted SV’s renal protective effects, preserving renal function in patients with chronic kidney disease [19]. This is particularly relevant for patients with SSc, who are at risk of renal impairment either from the disease itself, as in scleroderma renal crisis, or from cardiovascular complications.

## 5. Limitations

Although the study results are promising, this study has several limitations. This study’s small sample size and retrospective design have the potential for bias and reduce the generalizability of the results. Additionally, the cardiac-related data points were limited, as advanced imaging modalities, such as cardiac MRI and myocardial strain assessments, were not consistently available. Furthermore, there was no control group to show whether SV truly affected mortality and hospitalizations. Prospective observational studies or randomized controlled trials comparing SV to placebo or ACE-inhibitor–based GDMT in patients with SSc-related HFrEF can be beneficial in determining the benefits in this specific patient population, with an emphasis on evaluating SV’s potential benefits for other disease manifestations, including Raynaud’s phenomenon, pulmonary arterial hypertension, RV systolic function, and scleroderma renal crisis. By inhibiting neprilysin, increasing levels of vasoactive peptides, and promoting smooth muscle relaxation, SV may alleviate digital ischemia in RP and reduce pulmonary vascular resistance in PAH, potentially offering a therapeutic advantage in these SSc complications. For scleroderma renal crisis (SRC), the rationale for investigating SV lies in its mechanism of action, which involves dilating both the afferent and efferent arterioles. This dual effect reduces glomerular capillary pressure, a key driver of SRC pathophysiology.

## 6. Conclusions

Our findings suggest that SV could be a beneficial therapeutic option for patients with SSc and HFrEF, as it may help reduce the significant morbidity and mortality associated with heart failure by improving cardiac function and preserving renal function.

## Figures and Tables

**Table 1:** Baseline patient clinical characteristics and comparison between echocardiographic parameters, cardiac biomarkers, and clinical outcomes before and after S/V treatment.

Baseline Characteristics				All Patients (N = 24)		
**Female Gender**				18 (75%)		
**Age at diagnosis (y); mean (SD)**				53 (13)		
**Disease duration prior to S/V use (y); mean (SD)**				6.8 (5.6)		
**Scleroderma phenotype:**				N = 23		
- **Limited cutaneous**				11 (45%)		
- **Diffuse cutaneous**				9 (37%)		
- **Sine scleroderma**				1 (4%)		
- **Overlap**				2 (8%)		
**Duration of SV use (months); mean (SD)**				20.6 (20)		
**Indication for use:**						
- **HFrEF**				21 (87.5%)		
- **HFpEF**				3 (12.5%)		
**Heart failure etiology**						
- **Ischemic cardiomyopathy**				3(12.5%)		
- **Non-ischemic cardiomyopathy**				21(87.5%)		
**S/V discontinued**				6 (25%)		
**Reason for discontinuation:**				N = 6		
- **Hypotension**				4 (16%)		
- **Death**				2 (8%)		
**S/V dosage:**						
- **24/26 mg**				17 (70.8%)		
- **49/51 mg**				4 (16.7%)		
- **97/103 mg**				3 (12.5%)		
**Clinical, Lab, and Echocardiogram Data at Baseline and Follow-up**						
	N	Baseline	Follow-up	Δ	95% CI	*p*-value
**Systolic blood pressure (mmHg); mean (SD)**	22	128(20)	114(18)	−14(13)	(−20.1, −8.4)	<0.001
**LVEF (%); mean (SD)**	19	40.3(12.8)	47.7 (9.6)	7.4 (11.9)	(1.7, 13.2)	0.014
**RVSP (mmHg); mean (SD)**	11	41.2 (10.5)	37.3 (9.8)	−3.9 (9.5)	(−10.2, 2.5)	0.202
**NT-ProBNP; mean (SD)**	14	15,130.1 (23,394.3)	5082.4 (9221.8)	−10,047.7 (17,001.6)	(−19,864.1, −231.3)	0.046
**Creatinine (mg/dL); mean (SD)**	21	1.9 (3.6)	1.3 (0.9)	0.138 (0.306)	(−0.045, 0.260)	0.057
**Clinical outcomes**						
**Time to hospitalization (months); median**				64.4		
**Survival time (months); median**				52.9		

Results were represented as numbers (%) or mean ± standard deviation. y: Year; SD: Standard deviation; S/V: Sacubitril/valsartan; HFrEF: Heart failure with reduced ejection fraction; HFpEF: Heart failure with preserved ejection fraction; LVEF: Left ventricular ejection fraction; NT-ProBNP: N-terminal pro-B-type natriuretic peptide.

**Table 2:** Comparison between baseline and follow-up clinical, TTE, and laboratory parameters.

	N	Baseline	Follow-Up	Δ	95% CI	*p*-Value
**Clinical Parameters**						
**Systolic Blood Pressure (mmHg); mean (SD)**	22	128 (20)	114 (18)	−14 (13)	(−20.1, −8.4)	**<0.001**
**TTE Parameters**						
**LVEF (%); mean (SD)**	19	40.3(12.8)	47.7 (9.6)	7.4 (11.9)	(1.7, 13.2)	**0.014**
**LVESV (mL)**	8	83.8 (41.1)	73.4 (41.1)	−10.4 (16.7)	(−24.3, 3.6)	0.123
**LVESD (mm)**	13	39.5 (6.7)	38 (7.2)	−1.5 (4.3)	(−4.1, 1.2)	0.247
**LVEDV (mL)**	8	130.3 (50.9)	124.5 (47.7)	−5.8 (29.9)	(−30.8, 19.3)	0.604
**LVEDD (mm)**	16	50.4 (6.7)	49.6 (6.4)	−0.8 (6)	(−3.9, 2.4)	0.622
**LA Biplane Max Volume (mL)**	4	76.3 (19.8)	70.3 (24.1)	−6 (8.1)	(−18.9, 6.9)	0.234
**LA Biplane Volume Index; mean (mL/m^2^)**	6	37.4 (7.1)	36 (9.6)	−1.4 (5.9)	(−7.6, 4.8)	0.59
**RVSP (mmHg); mean (SD)**	11	41.2 (10.5)	37.3 (9.8)	−3.9 (9.5)	(−10.2, 2.5)	0.202
**RA Basal; mean (mm)**	5	41.4 (9.6)	39.6 (10.9)	−1.8 (5.8)	(−9.0, 5.4)	0.523
**RA Mid; mean (mm)**	4	28.8 (9.9)	29.8 (13.5)	1 (9.1)	(−13.5, 15.5)	0.841
**RV Base-Apex Length (mm)**	2	72.5 (12)	70.5 (12)	−2 (24)	(−218, 214)	0.925
**TAPSE; mean (cm)**	6	1.9 (0.4)	1.7 (0.5)	−0.2 (0.4)	(−0.6, 0.2)	0.239
**Tricuspid Regurgitant Velocity; mean (m/s)**	6	2.8 (0.4)	2.8 (0.5)	0 (0.3)	(−0.3, 0.3)	1.000
**LVOT Cardiac Output; mean (L/min)**	8	4 (1.3)	4.5 (1.2)	0.4 (1.6)	(−0.9, 1.8)	0.477
**LVOT Cardiac Index (L/min/m^2^)**	8	2.2 (0.7)	2.4 (0.5)	0.2 (0.7)	(−0.4, 0.8)	0.435
**Stroke Volume (mL)**	9	57.2 (15)	66.8 (20.2)	9.6 (27.7)	(−11.8, 30.9)	0.332
**Stroke Volume Index (mL/m^2^)**	9	32.7 (7.7)	37.9 (9.9)	5.2 (14.3)	(−5.7, 16.2)	0.304
**Labs**						
**NT-ProBNP; mean (SD)**	14	15,130.1 (23,394.3)	5082.4 (9221.8)	−10,047.7 (17,001.6)	(−19,864.1, −231.3)	**0.046**
**Creatinine (mg/dL);** **mean (SD)**	21	1.9 (3.6)	1.3 (0.9)	0.138 (0.306)	(−0.045, 0.260)	0.057
**Clinical Outcomes**						
**Time to Hospitalization (months); median**				64.4		
**Survival Time (months); median**				52.9		

Results were represented as mean ± standard deviation. TTE: Transthoracic echocardiogram; LVEF: Left ventricular ejection fraction; LVESV: Left ventricular end-systolic volume; LVESD: Left ventricular end-systolic dimension; LVEDV: Left ventricular end-diastolic volume; LVEDD: Left ventricular end-diastolic dimension; LA: Left atrium; RVSP: Right ventricular systolic pressure; RA: Right atrium; RV: Right ventricle; LVOT: Left ventricular outflow tract; NT-ProBNP: N-terminal pro-b-type natriuretic peptide.

**Table 3:** Comparison of scleroderma variants in patients treated with sacubitril/valsartan.

	Diffuse Scleroderma (N = 9)	Limited Scleroderma (N = 11)	95% CI	*p*-Value
**Female gender**	6 (66.7%)	8 (72.7%)		0.769
**Age at diagnosis (y); mean (SD)**	52 (19.2)	54.9 (10.9)	(−13.9, 19.7)	0.680
**Duration of S/V use; mean (SD)**	14.3 (15.7)	19.1 (16.6)	(−11.9, 21.5)	0.549
**S/V discontinued**	2 (22.2%)	4 (36.4%)		0.492
**Baseline LVEF (%); mean (SD)**	(N = 8)	(N = 7)		
	33 (9.2)	45.4 (14.1)	(−1.4, 26.3)	0.061
**Follow-up LVEF (%); mean (SD)**	(N = 8)	(N = 7)		
	43.5 (9.6)	51 (10.1)	(−3.5, 18.5)	0.163
**5-year survival events (N)**	(6)	(6)		
	44.4%	36.4%		0.9

Results were represented as numbers (%) or mean ± standard deviation. y: Year; SD: Standard deviation; S/V: Sacubitril/valsartan; LVEF: Left ventricular ejection fraction.

## Data Availability

The original contributions presented in this study are included in the article. Further inquiries can be directed to the corresponding author(s).

## Abbreviations

The following abbreviations are used in this manuscript:

SSc	Systemic Sclerosis
SV	Sacubitril/Valsartan
HFrEF	Heart Failure with Reduced Ejection Fraction
CMR	Cardiac Magnetic Resonance Imaging
ARNI	Angiotensin Receptor–Neprilysin Inhibitor
NT-proBNP	N-Terminal Pro-B-Type Natriuretic Peptide
LVEF	Left Ventricular Ejection Fraction
NYHA	New York Heart Association
REDCap	Research Electronic Data Capture
ICD	International Classification of Diseases
ACR/EULAR	American College of Rheumatology/European Alliance of Associations for Rheumatology
SD	Standard Deviation
HFpEF	Heart Failure with Preserved Ejection Fraction
RVSP	Right Ventricular Systolic Pressure
LA	Left Atrium
RA	Right Atrium
LVESV	Left Ventricular End-Systolic Volume
LVESD	Left Ventricular End-Systolic Dimension
LVEDV	Left Ventricular End-Diastolic Volume
LVEDD	Left Ventricular End-Diastolic Dimension
TAPSE	Tricuspid Annular Plane Systolic Excursion
LVOT	Left Ventricular Outflow Tract
mPAP	Mean Pulmonary Arterial Pressure
FDA	Food and Drug Administration
GDMT	Guideline-Directed Medical Therapy
SRC	Scleroderma Renal Crisis
RP	Raynaud’s Phenomenon
PAH	Pulmonary Arterial Hypertension
CI	Confidence Interval
pg/mL	Picograms per Milliliter
mg/dL	Milligrams per Deciliter
L/min	Liters per Minute
mL/m^2^	Milliliters per Square Meter
mmHg	Millimeters of Mercury
